# Residual-Free Micro–Nano Titanium Surfaces via Titanium Blasting and Single Acid-Etching: A Cleaner Alternative

**DOI:** 10.3390/bioengineering12070735

**Published:** 2025-07-05

**Authors:** Artiom Lijnev, José Eduardo Maté Sánchez de Val, Jeevithan Elango, Carlos Pérez-Albacete Martínez, José Manuel Granero Marín, Antonio Scarano, Sergio Alexandre Gehrke

**Affiliations:** 1 Department of Biomaterials Engineering, Faculty of Health Sciences, Universidad Católica de Murcia UCAM, Campus de los Jerónimos 135, 30107 Murcia, Spain; alijnev@ucam.edu (A.L.); jelango@ucam.edu (J.E.); cperezalbacete@ucam.edu (C.P.-A.M.); 2 Department of Implant Dentistry, Faculty of Medicine and Dentistry, UCAM-Universidad Católica San Antonio de Murcia, 30107 Murcia, Spain; jmgranero@ucam.edu; 3 Department of Innovative Technologies in Medicine & Dentistry, University of Chieti-Pescara, 66013 Chieti, Italy; ascarano@unich.it; 4 Department of Implantology, Bioface/Postgrados en Odontología/Universidad Católica de Murcia, Montevideo 11100, Uruguay

**Keywords:** implant surface, micro and micro–nano surface, in vitro study, osteoblasts

## Abstract

Background: Traditional sandblasted large-grit acid-etched (SLA) surface treatments frequently utilize alumina (Al_2_O_3_) blasting, which may leave residual particles embedded in implant surfaces, potentially compromising biocompatibility and osseointegration. This study investigates a contamination-free alternative: titanium dioxide particle (TiO_2_) blasting followed by hydrochloric acid (HCl) etching, aimed at generating a cleaner, hierarchical micro–nano-textured surface. Methods: Grade IV titanium disks were treated either with TiO_2_ sandblasting alone or with an additional HCl etching step. Surfaces were analyzed via atomic force microscopy (AFM), scanning electron microscopy (SEM), contact angle measurements, and profilometry. hFOB osteoblasts were cultured to assess adhesion, proliferation, metabolic activity, and morphology. Results: The combination treatment produced a more homogeneous micro–nano structure with significantly increased roughness and a cleaner surface chemistry. Osteoblast proliferation and metabolic activity were notably improved in the TiO_2_ and HCl group. SEM imaging showed a more organized cytoskeletal structure and pronounced filopodia at 72 h. Conclusions: Titanium blasting combined with HCl etching yields a cost-effective, contamination-free surface modification with promising early-stage cellular responses. This approach represents a safer and effective alternative to conventional SLA treatment.

## 1. Introduction

The long-term clinical success of dental implants is fundamentally dependent on effective osseointegration, defined as the direct structural and functional connection between living bone and the implant surface [1]. A key determinant of this process lies in the surface topography and chemistry of the implant, which together govern early protein adsorption, cell adhesion, proliferation, and subsequent tissue integration [2].

The clinical relevance of implant surface quality lies in its direct influence on the biological cascade following implantation. Immediately upon placement, the implant surface interacts with blood proteins, which condition the surface and mediate subsequent cellular events [2,3]. The micro- and nano-scale topography, together with surface chemistry, play a decisive role in modulating cell behavior, including adhesion, spreading, proliferation, and differentiation—particularly of osteoblasts, which are key for bone regeneration and osseointegration [4,5]. Surfaces with controlled roughness and high surface energy have been shown to enhance initial cell attachment and promote cytoskeletal organization and signaling pathways that favor proliferation and maturation [5,6]. Clinically, faster and more robust cell proliferation can translate into improved bone-to-implant contact (BIC), reduced healing times, and greater long-term implant stability [7]. Therefore, designing surfaces that optimize these cellular responses is critical for improving implant success rates, especially in patients with compromised bone quality.

Over the last two decades, the SLA technique has become one of the most widespread surface modification strategies due to its proven ability to create a hierarchically roughened topography that supports osteoblast activity and bone–implant contact [8,9,10]. SLA surfaces are typically generated using large-grit alumina (Al_2_O_3_) particle blasting, followed by double acid etching, commonly with HCl and sulfuric acid (H_2_SO_4_). While this method has shown clinical efficacy, several important limitations have emerged, both from a biological and industrial perspective [11,12,13].

First, a growing body of evidence suggests that alumina blasting leaves residual particles embedded in the implant surface [14]. These remnants are not bioinert and may elicit undesirable foreign body responses, including localized inflammation, fibrous encapsulation, or compromised bone apposition [15,16,17]. Even after ultrasonic cleaning or additional acid treatments, complete removal of Al_2_O_3_ contamination is often unachievable [14,18].

Second, the use of dual acid-etching protocols, while effective in producing nano-scale features and enhancing surface energy, introduces toxic reagents, notably H_2_SO_4_, into the manufacturing workflow. This not only elevates the cost and hazard level but the removal of H_2_SO_4_ from the process simplifies production and reduces chemical management requirements. It may also lead to non-uniform etching and variability between batches [19,20]. Additionally, H_2_SO_4_ introduces stronger corrosive effects, potentially impacting the underlying titanium integrity or increasing ion release [21].

In light of these drawbacks, alternative surface treatments that eliminate contamination risks, simplify acid processing, and promote more sustainable manufacturing practices are increasingly being explored. One such strategy is titanium particle blasting, which uses biocompatible TiO_2_ particles instead of alumina. As titanium is the same element as the base substrate, any residual particles do not compromise biocompatibility or trigger inflammatory cascades [18]. Furthermore, replacing dual acid etching with a single-step etch using HCl provides a safer, cleaner, and more cost-efficient method for generating nano-scale topography while maintaining high biological performance [22].

This study aims to evaluate a surface treatment approach that combines TiO_2_ particle blasting with single-step HCl etching. The objective is to establish whether this method offers a chemically clean, biocompatible, and topographically optimized surface capable of enhancing early osteoblastic responses relevant to osseointegration.

## 2. Materials and Methods

### 2.1. Sample Preparation

A total of 40 disks were manufactured by Implacil/Osstem (São Paulo, Brazil) using the same grade IV pure titanium employed in the production of dental implants. The manufacturing process adhered to the ASTM F67 standard established by the American Society for Testing and Materials (ASTM) [23]. Two experimental groups were established (n = 20 disks per group):

Surface 1 Group—The implants were subjected to sandblasting with commercially available TiO_2_ microparticles (150 ± 10 μm). Blasting was performed under 0.7 MPa pressure, through a 5 mm nozzle at a fixed distance of 10 mm, perpendicular (90° incidence), for 20 s per disk, using circular sweeping motions to ensure uniform coverage. These parameters were selected based on internal pilot tests and supported by the literature, which reports that TiO_2_ particle sizes between 106 and 180 μm at pressures of 0.6–0.8 MPa produce moderate-to-high micro-roughness (Sa ≈ 0.7–1.3 μm), ideal for osteoblastic activity [24]. For instance, blasting with 106–180 μm TiO_2_ particles yielded Sa ≈ 1.30 μm, achieving enhanced osteoblast proliferation and differentiation [25]. The chosen pressure and nozzle distance provide sufficient kinetic energy to reproducibly deform the titanium surface without inducing delamination or excessive crater formation.

Surface 2 Group—The implants were subjected to the same sandblasting procedure as the Surface 1 group, after which they underwent acid conditioning with 35% HCl, leading to a surface referred to as Superiore (Implacil/Osstem, São Paulo, Brazil).

**Figure 1:**
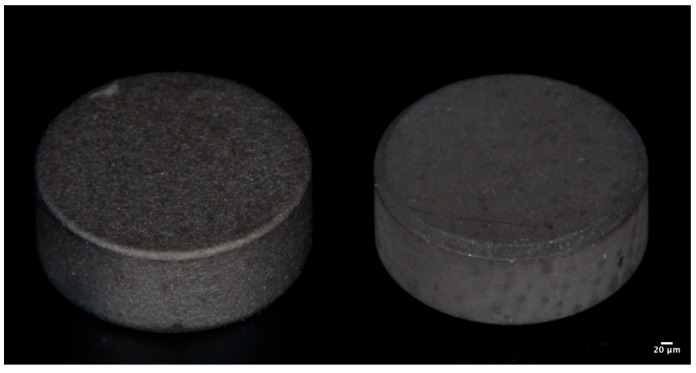
Representative image of the disks used: (**left**) surface treated with TiO_2_ sandblasting; (**right**) surface treated with combined treatment TiO_2_ and HCl.

All disks, each measuring 5 mm in diameter and 2 mm in height, were subjected to the same washing, decontamination, and sterilization processes as those applied to commercially available dental implants (Figure 1).

For experimental procedures, the number of disks per group used was as follows: Surface characterization (n = 8), in vitro cell culture (n = 12).

### 2.2. Surface Characterization Method

#### 2.2.1. Surface Topography and Morphology Method

An atomic force microscope (AFM, NaniteAFM, Nanosurf, Bracknell, Great Britain) was used to study the surface topography. The examinations were performed by tapping-mode at random sites area of (50 μm^2^) using a 5 μm^2^ scan head at a scan rate of <1 Hz. During the scanning process, several parameters were adjusted to achieve an enhanced image resolution. Furthermore, for the determination of the recorded roughness average (R_a_), the average maximum height of the profile (R_z_), mean root square roughness (R_q_), and maximum peak-to-valley roughness (R_max_) were also measured.

Further roughness measurements were conducted using a stylus profilometer (KLA Alpha Step D500). A 5-μm radius diamond cone stylus tip was used to analyze the surface held at 90° to the surface with a contact force range of 0.03–15 mg, a maximum height range of 1200 μm, and a 30 mm scan length. The disks were positioned at a 90° angle to the profilometer’s direction of travel, and four consecutive measurements were taken to estimate the roughness (Ra), utilizing the number average roughness for the calculations.

The disks’ surfaces were observed using scanning electron microscopy in a (SEM-Hitachi S-3500N) with an LED detector, at a 5 kV acceleration voltage. The disks were oriented both horizontally and vertically in relation to the detector and were captured at various magnifications: 1 K (10 μm), 6 K (2 μm) for the horizontal position and 6 K (2 μm), 8 K (4 μm) for the vertical position.

#### 2.2.2. Contact Angle Test

Water contact angle measurements were carried out by the falling drop method using a contact angle instrument (Contact Angle Goniometer, Zeiss, Oberkochen, Germany) at room temperature (with an RH of 60%). Briefly, 5 µL of deionized water was dropped on the surface of the disks, and the contact angle was measured at 10 s time points. The formed contact angle between the tangent at the liquid–disk interface was recorded.

### 2.3. In Vitro Cell Culture Test

The human osteoblast cell line (hFOB) (ATCC-CRL-3602, LGC Standards, Barcelona, Spain, Order Ref. No. 70060968) was used to evaluate the biocompatibility of the samples. The cells were cultured according to the handling procedure information provided by American Type Culture Collection (ATCC) using complete Ham’s F12 Dulbecco’s Modified Eagle’s Medium (Thermofisher catalog # 21041-025), consisting of 10% Fetal Bovine Serum (FBS) and 0.3 mg/mL of Geneticin (G-418 Sulfate) (Thermofisher catalog #10131027) in a 5% CO_2_ incubator at 34 °C. Once the cells reached approximately 80–90% confluence, the medium was discarded, and the cells were gently rinsed with phosphate-buffered saline (PBS) to eliminate any residual medium or unbound cells. Trypsinization was performed by adding 2 mL of 0.25% (*w*/*v*) trypsin-0.53 mM EDTA to a T75 flask, ensuring cell layer dispersion under an inverted microscope. The isolated cells were subjected to centrifugation, and the resulting pellet was resuspended in fresh medium for cell passage. Each time, the cell count was determined using an automated Invitrogen cell counter (Countess 3 FL, Thermo Fisher Scientific, Waltham, MA, USA). The passages of cells utilized for this experiment varied from passage 2 to 4.

#### 2.3.1. Proliferation Assay

The proliferation of hFOB cells cultured in surface 1 and surface 2 samples was evaluated at 24 and 72 h. Cells were initially seeded at a density of 5 × 10^4^ on the specimens and incubated for 3 h, after which the specimens were covered with medium. Following the specified incubation periods, the cells were trypsinized, as previously described, and 10 mL of the cell suspension was transferred to separate Eppendorf tubes and stained with 10 mL of trypan blue. The total cell count and viability were then evaluated using an automated Invitrogen cell counter. Cells that were seeded without any specimens served as the control group.

#### 2.3.2. Cytotoxicity Method

For cell metabolic activity assessment, the cells were seeded and cultured on the disk’s surface, as described previously. The effect of surface treatment was determined by using the 3-(4,5-dimethylthiazol-2-yl)-2,5-diphenyltetrazolium bromide (MTT) assay. Briefly, 50 µL of MTT reagent (5 mg/1 mL PBS) with fresh medium was added to each well at the previously established time point and incubated for 3 h. Then, the MTT solution was removed, and 150 µL of DMSO was added to each well. The formed formazan crystal was quantified spectrophotometrically at 570 nm using a SpectraMax iD3 Multi-Mode Microplate reader (Molecular Devices, LLC., San Jose, CA, USA). The cell cultures without specimens were regarded as the control.

#### 2.3.3. Cell Morphology Test

Cell morphology analysis was performed by SEM. The cells were cultured on disks with a density of 5 × 10^4^, following previously described methods, and incubated for 24 and 72 h. The unbonded cells were removed by rinsing with PBS, dehydrated in a series of ethanol, and fixed with 4% paraformaldehyde (PFA). Disks containing fixed cells were stored in PBS until the critical point drying process. During this phase, the cells were dehydrated in a CO_2_ environment to preserve their morphology. Subsequently, the disks were coated with a layer of gold and observed under SEM (SEM-Hitachi S-3500N, Hitachi, Tokyo, Japan) with magnifications of 1 K (10 μm), 3 K (2 μm).

Additionally, a new set of disks with cells were examined to observe adhesive conditions on the specimens using an optical microscope (Leica, Leica Microsystems, Wetzlar, Germany), coupled with digital camera and fluorescence filters (xxx). Disks containing cells that had been fixed for 24 and 72 h, as previously outlined, were washed twice with PBS and then permeabilized by the addition of Triton X-100 (1 mL per 100 mL of PBS) for a duration of 10 min. The actin cytoskeleton was labeled using Phalloidin-FITC, while DAPI was employed to stain the nuclei.

### 2.4. Statistical Analyses

All the experiments were conducted in independent setups, and the results were obtained in triplicate. The data were presented as the mean standard deviation (SD). Normality and equal variance tests were performed prior to the testing. Different data groups were compared using an ANOVA test. Statistical significance was considered at a probability less than 0.05 (*p* < 0.05) using SPSS 29.9 (IBM Corp., Armonk, NY, USA).

## 3. Results

### 3.1. Surface Characterization Results

#### 3.1.1. Surface Topography and Morphology Results

**Figure 2:**
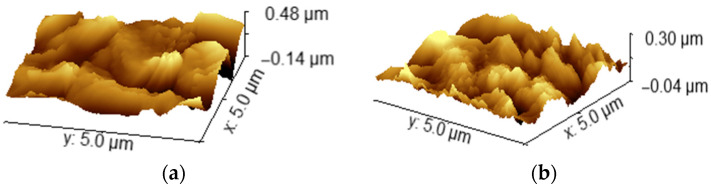
Atomic force microscopy (AFM) of titanium surfaces used in the study: (**a**) surface treated with TiO_2_ sandblasting; (**b**) surface treated with combined treatment TiO_2_ and HCl.

Surface modification via HCl treatment induced notable changes in the nano-scale topography of grade IV titanium disks. AFM imaging confirmed that both groups exhibited rough surfaces; however, the nano-scale roughness observed in sandblasted disks was noticeably reduced compared to the combined treatment. The latter displayed distinct differences in waviness, suggesting enhanced surface complexity (Figure 2).

The combined treatment group exhibited more pronounced alterations, resulting in increased surface roughness and non-uniform topography. Both nano- and micro-scale Ra values showed significant increases, and comparative analysis revealed significant differences in Ra between groups, as measured by AFM (*p* < 0.05) and stylus profilometry (*p* < 0.001). However, AFM R_z_ values showed no significant differences between groups. In contrast, the R_q_ parameter demonstrated a statistically significant difference (*p* = 0.02), while no significant differences were observed in R_max_ (Table 1). Representative stylus profilometer profiles are presented previously in Figure 2. The variability is likely due to the heterogeneous nano-scale texture introduced by blasting and acid propagation, which creates localized peaks and valleys across different measurement points.

**Figure 3:**
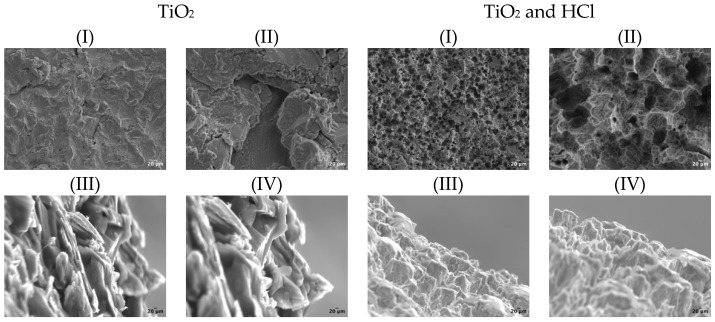
Schematic representation of SEM inspection images illustrating horizontal and vertical surface morphology across different surface treatments at various magnifications (I) 1 K; (II) 6 K; (III) 6 K; (IV) 8 K. The horizontal view presents a top-down perspective of the disk surface, highlighting the overall distribution of surface features such as pores and microstructures. The vertical view shows a cross-sectional-like morphology that reveals the depth and contours of the surface irregularities, providing insight into the roughness and profile variation created by different surface treatments.

SEM inspection revealed distinct surface patterns in both horizontal and vertical orientations. At lower magnifications, the combined treatment group exhibited a greater density of uniformly distributed micro-pits across the surface, whereas the sandblasted group with sandblasting alone displayed a more geometric pattern with crystal-like structures, less uniform pits, and a more consistent surface texture (Figure 3). These observations correlate with variations in R_a_ values between surface treatments. Upon closer examination, lacuna-like depressions were more pronounced in the combined treatment group, likely resulting from acid propagation into the titanium disk. In contrast, the sandblasting process, being more superficial, led to fewer pronounced depressions, alongside small, unsupported irregularities.

Vertical exploration revealed distinct surface characteristics between the treatment groups. The HCl-treated group exhibited lacuna-like depressions extending onto lateral protuberances, whereas the sandblasted-alone group displayed a more laminar surface morphology. Immersion in HCl consistently led to visibly more defined grain boundaries, an effect that became increasingly pronounced in lateral views compared to the sandblasting. An intriguing observation was the presence of cracks within certain protuberances, which were distinctly visible in lateral perspectives.

#### 3.1.2. Contact Angle Results

**Figure 4:**
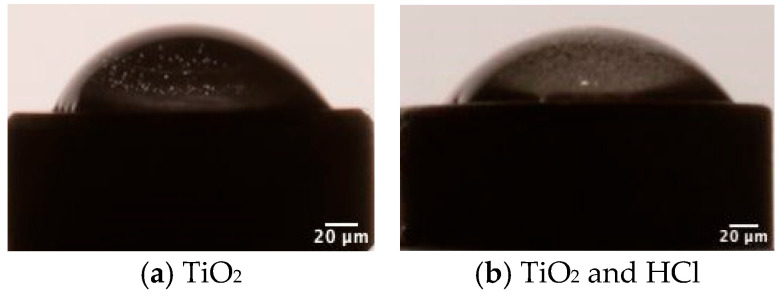
Schematic representation of the water contact angle of two surface treatment samples.

Surface wettability was measured by determining the water contact angles (Figure 4). After sandblasting, the surface of the disk became hydrophobic, as the mean contact angle reached the value of 45° ± 3. The combination of sandblasting and HCl was found to increase the hydrophilicity of the sample, with the angle value 40° ± 1, and there were no statistical differences found in water contact angle measurements (*p* > 0.05).

### 3.2. In Vitro Cell Culture Results

#### 3.2.1. Proliferation Results

**Figure 5:**
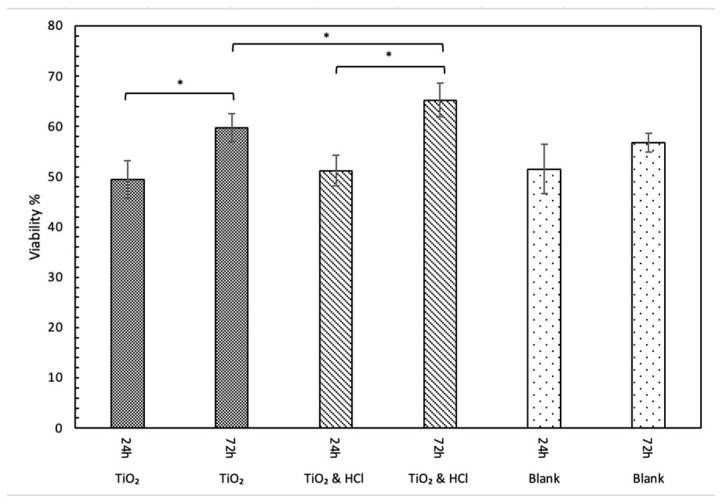
Cell viability represented as % in each group. Cell viability (%) of hFOB cells on TiO_2_ sandblasting and TiO_2_ and HCl-treated surfaces at 24 h and 72 h. A significant increase was observed over time in both groups, with higher viability in the acid-treated group at 72 h (*p* < 0.05). *: statistically significant difference (*p* < 0.05).

**Figure 6:**
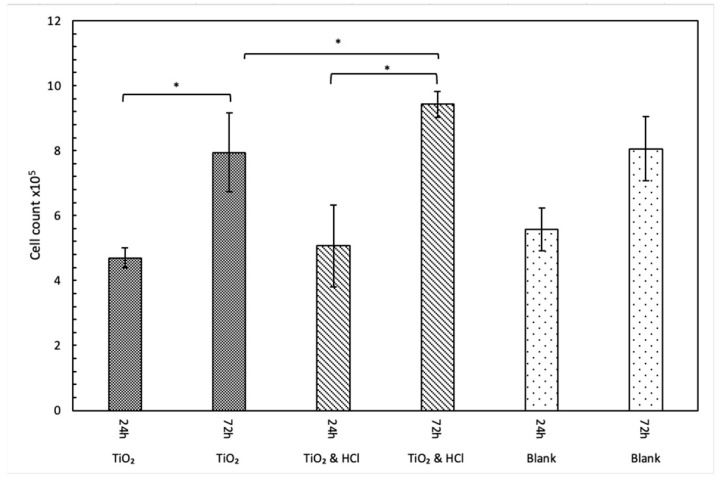
hFOB cell proliferation on TiO_2_ sandblasting and TiO_2_ and HCl surfaces at 24 h and 72 h. A time-dependent increase was observed, with the TiO_2_ and HCl group exhibiting significantly enhanced proliferation at 72 h compared to TiO_2_ sandblasting (*p* < 0.05). *: statistically significant difference (*p* < 0.05).

Viability and proliferation of hFOB on two surface-modified titanium disks at 24 and 72 h are shown in Figure 5 and Figure 6, respectively. Both groups exhibited a significant increase in cell proliferation from day 1 to day 3 (*p* < 0.001). During the initial 24 h, no statistically significant differences were observed between the groups (*p* = 0.09). However, by 72 h, the acid-enhanced treatment group demonstrated a notable increase in proliferation compared to both the sandblasted group (*p* = 0.02) and the control group, indicating a favorable effect of acid etching. Cell viability increased significantly in both groups from 24 to 72 h. Intergroup comparisons at 72 h revealed statistically significant differences, indicating variations in cellular response between the treatment conditions.

#### 3.2.2. Cytotoxicity Results

**Figure 7:**
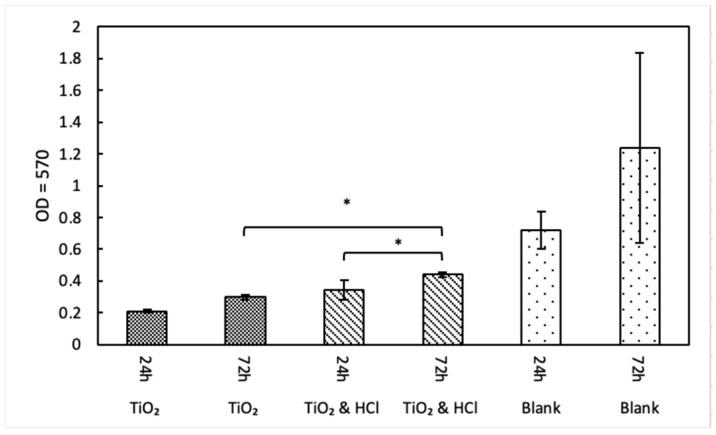
Metabolic activity of hFOB cells with different surface treatments, significant differences can be observed at day 3, favoring acid-enhanced groups. *: statistically significant difference (*p* < 0.05).

MTT analysis was conducted at 24 and 72 h of culture, revealing a significant increase in cell metabolic activity within the acid-enhanced group from day 1 to day 3. Additionally, a statistically significant difference was observed between the two groups at day 3 (*p* < 0.001), suggesting that metabolic activity may serve as an indirect indicator of cellular proliferation rate, with the acid-treated surface exhibiting superior cell response compared to the sandblasted group (Figure 7). When compared to the Blank (control), both surface-treated groups showed lower metabolic activity. The control group displayed the highest OD values at both time points but with greater variability, likely reflecting the absence of surface constraints and potential cell overgrowth.

#### 3.2.3. Cell Morphology Results

**Figure 8:**
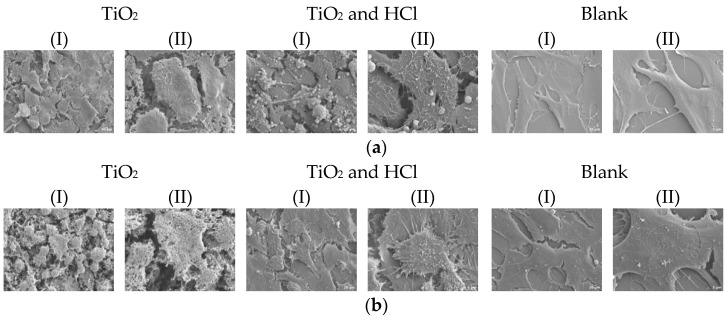
SEM cell morphology of disks during (**a**) 24 h and (**b**) 72 h, under different magnification (I) 1 K; (II) 3 K.

Cell morphology was qualitatively assessed via SEM after 24 and 72 h of culture. SEM images of both groups showed that both flattened and elongated hFOB cells adhered to the surfaces. Filopodia attachments were found on all samples; however, these attachments were more abundant on etched groups, especially after 72 h of culture. Sandblast showed reticular-shaped osteoblasts at 24 h, whereas at 72 h showed spindle-shaped hFOB. SEM images revealed that osteoblasts spread very flat and attach tightly to both surfaces of TiO_2_ sandblasting and TiO_2_ + HCl (Figure 8). Cells in the Blank group (no material) exhibited wide lamellar morphology with less directionality and greater variability, likely due to the absence of surface topographical cues.

**Figure 9:**
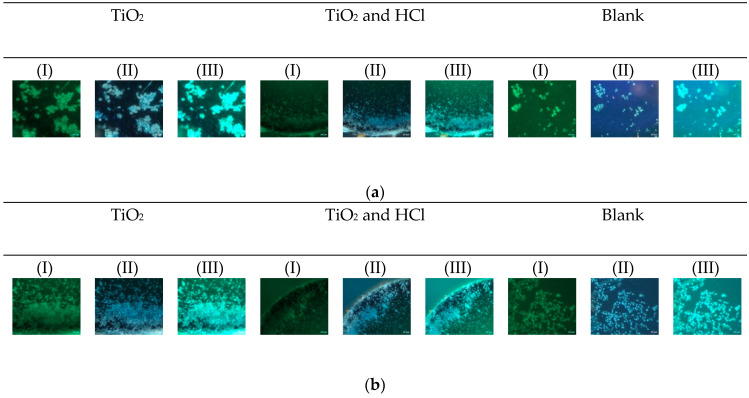
hFOB adhesion and cytoskeleton were evaluated on the surfaces after (**a**) 24 h and (**b**) 72 h, (I) FITC; (II) DAPI; (III) merged.

Cell shape has lately been considered an emerging property of the subtle interplay between cellular phenotype and physical properties. The fluorescence intensity and the number of hFOB cells were increased from 24 to 72 h (Figure 9). In general, the osteoblast presented circular morphology; however, the cell spreading area increased slightly with the acid treatment and exhibited many cytoplasmic extensions and filopodia. The Blank group presented larger and more irregularly distributed cells at both time points, with increased fluorescence intensity but less orientation.

## 4. Discussion

This study introduces a contamination-free surface treatment method for titanium implants using TiO_2_ particle blasting followed by HCl etching. Unlike the conventional SLA approach that uses alumina blasting, our method eliminates the risk of embedded aluminum particles, a concern increasingly raised in recent implant literature [12,15]. Similarly to other studies, we have observed that blasting with TiO_2_ particles does not leave detectable residual contamination on titanium implant surfaces, resulting in a clean surface. Additionally, we refer to this method as a “*cleaner alternative*” not only due to its ability to generate residual-free surfaces, but also because it simplifies the chemical processing by avoiding the use of sulfuric acid—a highly corrosive and hazardous reagent used in conventional dual acid SLA protocols. This streamlined process reduces both chemical risk and environmental burden, while improving reproducibility and safety during manufacturing. Therefore, the term “cleaner” encompasses both surface purity and process hygiene.

From a surface engineering perspective, the Superiore method achieves hierarchical micro–nano topography by combining two accessible and biocompatible techniques. The surface roughness analysis via AFM and stylus profilometry showed significant increases in both R_a_ and R_q_ in the TiO_2_ and HCl groups. The enhanced complexity of the surface provides anchorage sites for initial protein adsorption, promotes cell adhesion, and supports osteogenic differentiation. These parameters position the TiO_2_ and HCl surface within the range of what is considered a medium rough surface, a category that has been widely demonstrated to elicit superior biological behavior in terms of osteoblastic adhesion and bone–implant contact [26,27]. Similar findings in surface roughness metrics have been reported in more complex treatment procedures such as double acid etching. For instance, some authors have demonstrated that SLA type surfaces treated with HCl/H_2_SO_4_ achieved R_a_ values in the range of 1.5–2.0 µm and similarly high R_q_ values, placing the TiO_2_ and HCl surface within a comparable topographical spectrum [28,29].

Morphological SEM data indicated that acid etching post-blasting produced deeper lacunae and more defined grain boundaries. This aligns with reports indicating that acid-induced nano-scale porosity increases surface energy and closely replicates the structural characteristics of trabecular bone [19,30]. Although HCl-only treatment lacks the aggressive dual acid etching of SLA, its in vivo biological performance remained comparable, as also demonstrated by studies reporting favorable osseointegration outcomes in single acid-treated implants within preclinical models [22].

Indeed, one of the most remarkable findings of this study is that the TiO_2_ and HCl promoted statistically significant osteoblast proliferation and higher metabolic activity at 72 h when compared to Ti blasting alone, utilizing the human osteoblast cell line (hFOB). These findings support the hypothesis that single acid-etched surfaces can achieve biological responses comparable to those of dual acid-etched surfaces, particularly when preceded by effective surface roughening techniques like sandblasting.

Comparative studies support this observation, emphasizing that the addition of a second acid in dual treatments only marginally enhances nano-topography but does not guarantee superior biological outcomes [30]. Additional studies comparing various surface treatments have shown that they elicit similar osteoblastic responses, highlighting that the presence of a roughened microstructure often plays a more critical role than the specific chemical aggressiveness of the etching protocol [31].

Furthermore, the use of TiO_2_ particles instead of Al_2_O_3_ for blasting offers a crucial biocompatibility advantage. Titanium particles, being chemically identical to the implant substrate, do not introduce foreign materials that may interfere with cellular responses. Several studies have demonstrated that TiO_2_-blasted surfaces exhibit cleaner surface chemistry and lower inflammatory potential compared to Al_2_O_3_-blasted counterparts. This cleaner profile may enhance early cell adhesion and proliferation by reducing the presence of embedded contaminants that can impair protein adsorption and integrin signaling pathways [18].

Clinically, our findings suggest that the TiO_2_ and HCl surface modification may replicate the performance of SLA surfaces while avoiding its pitfalls. Traditional SLA surfaces, although effective, require handling multiple corrosive agents and may result in embedded contaminants. Our method, in contrast, uses a simplified single acid approach, eliminating the need for sulfuric acid, which not only reduces the chemical load and environmental impact but also facilitates better reproducibility and industrial scale-up.

From a manufacturing standpoint, TiO_2_ particles are more expensive than Al_2_O_3_, but the overall cost can be offset by eliminating H_2_SO_4_ processing, minimizing cleaning steps, and reducing equipment corrosion. This makes the TiO_2_ and HCl method not only safer and cleaner but also more cost-effective in the long term.

While dual acid treatments like HCl/H_2_SO_4_ can enhance nano-roughness and surface energy, their biological benefits appear marginal when effective micro-roughness is already achieved through blasting, which plays a more decisive role in promoting osteoblast behavior than the number of acids used [14,19,31].

Therefore, our results contribute to a growing consensus that the synergistic effect of mechanical roughening (via sandblasting) and chemical modification (via a single mild acid) is sufficient to trigger robust cellular responses, without the risks associated with dual acid protocols.

This study presents some limitations that should be acknowledged. First, the sand blasting parameters were optimized based on preliminary tests and literature data, but a more comprehensive parametric study could provide deeper insights into their influence on surface quality. Second, the analysis of surface characteristics was limited to selected techniques, and additional methods such as 3D profilometry or advanced microscopy could further elucidate the surface morphology. Third, the long-term performance of the treated surfaces under real environmental conditions was not evaluated, which restricts the understanding of durability and wear behavior. Finally, the study focused on a specific material and treatment protocol, so the results may not be directly generalized to other materials or blasting conditions without further investigation.

## 5. Conclusions

Within the limitations of this in vitro study, we conclude that the surface modification combining TiO_2_ particle blasting with single-step HCl etching represents a residual-free and biocompatible micro–nano surface for titanium implants. This treatment generated a hierarchical topography with increased roughness and promoted enhanced early osteoblast proliferation, metabolic activity, and cell morphology when compared to blasting alone.

Furthermore, this approach can be considered a “cleaner alternative” to conventional SLA treatments in two main aspects: (1) it results in a chemically cleaner surface, free from embedded contaminants such as alumina particles; and (2) it simplifies the surface treatment protocol by using only HCl instead of dual acid (HCl/H_2_SO_4_) etching, thereby reducing chemical handling risks, environmental impact and industrial complexity.

Thus, this method offers a safer, more reproducible, and potentially more sustainable surface modification strategy. Nonetheless, further in vivo validation is required to confirm the clinical relevance of these findings.

## Figures and Tables

**Table 1:** Average R_a_, R_q_, R_z_, and R_max_ values of titanium surfaces treated with sandblasting and sandblasting followed by acid conditioning (average ± SD). The ANOVA test revealed differences in the R_a_ (AFM, *p* < 0.05; SP, *p* < 0.001), R_q_ (AFM, *p* = 0.02) values of sandblasted followed by hydrochloric acid, compared to sandblasted samples.

	R_a_ (nm, AFM)	R_z_ (nm, AFM)	R_q_ (nm, AFM)	R_max_ (nm, AFM)	R_a_ (nm, SP)
TiO_2_	850.9 ± 133.2	7.7 ± 3.7	1.1 ± 3.2	10.1 ± 12.1	922 ± 134.0
TiO_2_ and HCl	921.2 ± 143.0 *	8.1 ± 2.8	1.2 ± 4.1 *	10.5 ± 16.2	1.315 ± 165.0 *

AFM: Atomic force microscopy; SP: Stylus profilometer; TiO_2_: Sandblasting with titanium particles; HCl: Hydrochloric acid; *: statistically significant difference (*p* < 0.05).

## Data Availability

The original contributions presented in this study are included in the article. Further inquiries can be directed to the corresponding authors.

## Abbreviations

The following abbreviations are used in this manuscript:

AFM	Atomic force microscopy
SEM	Scanning electron microscopy
SP	Stylus profilometer
hFOB	Human osteoblasts
